# Monitoring population salt intake using casual urinary sodium: Tehran Lipid and Glucose Study

**DOI:** 10.1186/s12986-022-00658-4

**Published:** 2022-03-15

**Authors:** Zahra Bahadoran, Parvin Mirmiran, Reza Norouzirad, Asghar Ghasemi, Fereidoun Azizi

**Affiliations:** 1 Nutrition and Endocrine Research Center, Research Institute for Endocrine Sciences, Shahid Beheshti University of Medical Sciences, Tehran, Iran; 2 Department of Biochemistry, School of Medicine, Dezful University of Medical Sciences, Dezful, Iran; 3 Endocrine Physiology Research Center, Research Institute for Endocrine Sciences, Shahid Beheshti University of Medical Sciences, No. 24, Sahid-Erabi St, Yemen St, Chamran Exp, P.O. Box, 19395-4763 Tehran, Iran; 4 Endocrine Research Center, Research Institute for Endocrine Sciences, Shahid Beheshti University of Medical Sciences, Tehran, Iran

**Keywords:** Casual urinary sodium, Estimated population-salt intake, Tanaka equation

## Abstract

**Background:**

We aimed to estimate salt intake among an Iranian population using spot urine-based equations and a dietary-based method.

**Methods:**

Adult men and women (n = 2069) were recruited from the Tehran Lipid and Glucose Study (2014–2017). Urinary sodium (Na), potassium (K), and creatinine (Cr) concentrations were measured in the morning spot urine samples. The 24-h urinary Na excretion and predicted salt intake was estimated using five equations, i.e., Kawasaki, Tanaka, Intersalt, Toft, and Whitton. A validated food frequency questionnaire (FFQ) was used to obtain dietary intake of salt. The agreement of each urinary- and FFQ-based salt estimation with the overall mean of the methods, considered as the gold standard, was assessed using the Bland–Altman method.

**Results:**

Mean age of the participants was 45.6 ± 14.8 y, and 45.4% were men. Mean (SD) estimated salt intake, derived from the overall mean of the methods, was 9.0 ± 2.2 g/d (10.2 ± 2.1 and 7.9 ± 1.7 g/d in men and women, respectively). Mean bias of the estimations from the overall mean ranged from − 0.2.42 to 2.75 g/d, with the Tanaka equation having the least bias (mean bias = 0.13 ± 1.10, 95% CI − 2.37, 2.30 g/d). Tanaka estimated a mean salt intake of 8.9 g/d (range 2.1 to 18.7 g/d); accordingly, only 5.1% of participants adhered to the recommendation (< 5 g/d salt intake), whereas 26.8% and 2.4% exceeded the recommendation by 2- and threefold.

**Conclusion:**

The Tanaka equation could provide a more accurate mean-population estimated salt intake from casual urinary Na concentration in our population. About 95% of the Iranian population exceeded the current recommendations of salt intake.

## Introduction

Cardiovascular diseases (CVD) and hypertension (HTN) are growing global health problems; in 2019, 523 and 18.6 million prevalent cases of CVD and CVD deaths and 33% global age-standardized prevalence of HTN in adults were reported [1, 2]. The first‐line antihypertensive agents are thiazide diuretics, beta-blockers, calcium channel blockers, angiotensin-converting enzyme (ACE) inhibitors, angiotensin II receptor antagonists, or alpha adrenergic blockers [3]. Complementary medicine and dietary approaches have also been proposed to manage CVD risk factors and HTN [4–7].

Excess salt intake is suggested to be responsible for a global 3 million deaths, and 70 million disability-adjusted life-years (DALYs) lost every year [8]. Current literature confirms the biological plausibility of the association between high intake of sodium (Na) and HTN and CVD events [9]. Although excessive Na intake remains undefined [10, 11], monitoring population salt intake is suggested as a global health priority [12, 13]. The World Health Organization (WHO) recommends less than 85 mmol/day of Na (< 2 g/d Na or ~ 5 g/d salt) for adults [14]. The Dietary Guidelines for Americans (DGA) recommends a reduction of Na intake to 100 mmol/day (< 2.3 g/day) and 65 mmol/day (1.5 g/day) in the general population and individuals with hypertension, diabetes, or chronic kidney disease [15]. The global estimation of mean intake of salt is around 2- to 3-fold higher than the current limit (10 g/d [16] to 14 g/d [8] of salt), and 95% of the World's population have a mean salt intake between 6 and 12 g/d [17].

Although the 24-h urine sampling is the gold standard for assessing Na excretion and estimating salt intake [18], the WHO considers spot urine sampling a less-burden and more feasible method of population salt intake monitoring [12]. Several equations have been developed to predict 24-h Na excretion from Na concentration of spot urine samples along with urinary creatinine and potassium (K) concentration, age, height, weight, and sex [19]; however, the validity and reliability of the equations are varied across populations due to diverse ethnicity and different patterns of Na intakes [19, 20]. Dietary methods tend to underestimate population salt intake [19, 21], and poor agreement is reported between estimated-Na from the food frequency questionnaire (FFQ) and 24‐h urine [22].

Choosing a valid method to monitor population salt intake over time as an alternative to more expensive and less feasible methods like 24-h urine collection or food-based estimation is now a research priority in the public health area. We aimed to estimate salt intake among an Iranian urban population using spot urine-based equations and the FFQ-based method.

## Material and methods

### Study population

This cross-sectional sectional study was conducted in the framework of the Tehran Lipid and Glucose Study (TLGS), an ongoing population-based cohort study initiated in 1999 on a representative sample of males and females aged ≥ 3 years to investigate and prevent non-communicable diseases [23]. For this study, adult men and women (age ≥ 19 years) were recruited from the sixth examination of the TLGS (2014–2017) and included in the analyses if they had completed measurements on spot urinary Na, K, and creatinine, as well as demographics, anthropometrics, biochemical measurements, and usual dietary intakes. Under- or over-reported daily energy intake (< 800 kcal/d or > 4200 kcal/d, respectively) were considered as exclusion criteria, and final analyses were conducted on 2069 subjects (940 men and 1129 women).

### Demographic and anthropometric measurements

Detailed measurements of the variables, including demographics and anthropometric measurements, were reported elsewhere [24]. In brief, body weight was measured using a digital scale (Seca, Hamburg, Germany) while the participant was minimally clothed, without shoes, and was reported to the nearest 100 g. Height was measured using a tape meter in a standing position without shoes and was reported to the nearest 0.5 cm. Body mass index (BMI) was calculated as weight (kg) divided by the square of the height (m^2^). Waist circumference was measured to the nearest of 0.1 cm at the level of the umbilicus, over light clothing, using a soft tape meter, and without any pressure to the body.

The physical activity was assessed using the Modifiable Activity Questionnaire (MAQ); the frequency and time spent on light, moderate, hard, and very hard intensity activities according to the list of everyday activities of daily life over the past year were documented and physical activity levels expressed as metabolic equivalent hours per week (MET-hour/week) [25]. Reliability and validity of the Persian version of the MAQ have previously been investigated [26].

Systolic (SBP) and diastolic (DBP) blood pressures were measured using a standard mercury sphygmomanometer calibrated by the Institute of Standards and Industrial Research of Iran [27]. Blood pressure was measured twice on the participants' right arm, after a 15-min rest in a sitting position, with at least a 30-s interval between two measurements. The two measurements’ mean was considered the participant's BP.

### Urine sampling and measurement of urinary metabolites

Casual urine samples were obtained between 7:00 and 9:00 AM following overnight fasting. Aliquots of the casual urinary samples were frozen and sent to the central laboratory of the TLGS. Urinary concentrations of Na and K were measured by flame photometry (Screen lyte, Hospitex Diagnostics, Florence, Italy). Intra- and inter-assay coefficients of variation (CVs) were ≤ 2.8% and 4.8% for Na and K, respectively. Spot urinary Cr concentrations were measured using the Jaffe method; both inter- and intra-assay CVs were ≤ 5%.

### Estimation of 24-h Na from the spot urine sample

Although the 24-h urine sampling is the gold standard for estimation of Na intake [18], it is criticized for potential bias due to under- and over-collection of samples, imposing considerable burden for participants, and low response rates (~ 10–40%), which affects data collection in representative population-based studies [21, 28]. In contrast, spot urine sampling has received much recent attention because it can easily incorporate into population-based settings without potential inaccuracy of sample collections [21, 28].

Due to the lack of an accurate and reliable predictive equation developed explicitly for our population, we applied the most commonly used and validated equations to estimate 24-h urinary excretion (mg/d) from Na concentration (mmol/L) of spot urine sample, including Kawasaki [29], Tanaka [30], Intersalt (also includes spot urine K concentration) [31], Toft [32], and Whitton [20]. Table 1 provides details of the equations with a brief description. The Kawasaki, Tanaka, and Whitton equations have been developed among Asian populations, while Intersalt and Toft equations have been developed among Western populations [20, 29–32].

The values of Na intake (mg/d) were converted to salt intake (g/d) by multiplying the value of Na excretion (mg/d) by 0.00254 (2.54 ÷ 1000). The estimated 24-h urinary excretion from a single spot urine sample provided the Na status of the participants in a single day.

### Dietary assessment

The usual dietary intakes of the participants over the previous year were assessed using a validated semi-quantitative 147-item FFQ. Details of dietary assessment in the TLGS were described elsewhere [33]. In brief, the frequency of food items consumed during the past year was asked daily, weekly, or monthly. Portion sizes of consumed foods reported in household measures were converted to the gram. Since the Iranian Food Composition Table is incomplete and has limited data on raw foods and beverages' nutrient content, the US Department of Agriculture Food Composition Table was used [34]. Usual dietary intake of Na and K were also obtained from nutritional data and are reported as mmol/d. The FFQ provided the mean intake of Na and K of the participants over the last year.

### Statistical methods

Statistical analyses were conducted using SPSS for Windows version 20 (SPSS Inc., Chicago, IL, USA) and the GraphPad Prism version 8.00 for Windows (GraphPad Software, CA, USA). A two-tailed *P* value < 0.05 was considered statistically significant. Dietary intakes of Na and K were adjusted for total energy intake using the residuals’ method [35]. Mean, and standard deviation (SD) of values and the frequency (%) of characteristics of the participants were compared between men and women using an independent sample t-test or Chi-square test.

Due to the lack of accessibility to 24-h urine samples, as the gold standard, we compared each estimation with the mean of all estimations (spot urine-based equations and FFQ-based estimation). In the case of lacking a gold standard method for method comparison, mean of available assay methods can be considered as the reference [36, 37].

Bland–Altman difference plots were used to assess the agreement between each assay and the overall mean (i.e., provided as estimated mean bias and 95% CI). The regression equation (slope and intercept) for bias [difference of each estimation as dependent variable (y) *vs.* overall mean of 6 estimations as independent variables (x)] was determined using least squared perpendicular distance regression analysis (Deming’s method) [38], which is preferred over ordinary linear regression (OLR) for method comparison studies in which both variables are measured with error [39]. In OLR, it is assumed that random error, arising from inherent limitations of measurements, is constant over the range of the data whereas, in the Deming’s regression, random errors of both compared method are taken into account [40]. In Deming’s regression, both x and y variables are subject to error, and the squares of the perpendicular distances of the x and y points from the regression line are minimized [38].

## Result

The mean age of the participants was 45.6 ± 14.8 y, and 45.4% were men. Table 2shows the characteristics of the study participants. Mean casual urinary Na concentration was 135 ± 56.5 mmol/L (143 ± 54.9 and 127 ± 56.8 in mmol/L, in men and women, respectively, *P* < 0.05); urinary Na-to-K ratio was higher in men compared to women (2.39 ± 1.45 vs. 2.19 ± 1.34, *P* < 0.05). The mean estimated Na intake was 144 ± 36.3 mmol/d (142 ± 37.3 and 145 ± 35.4, in men and women, respectively).

**Fig. 1:**
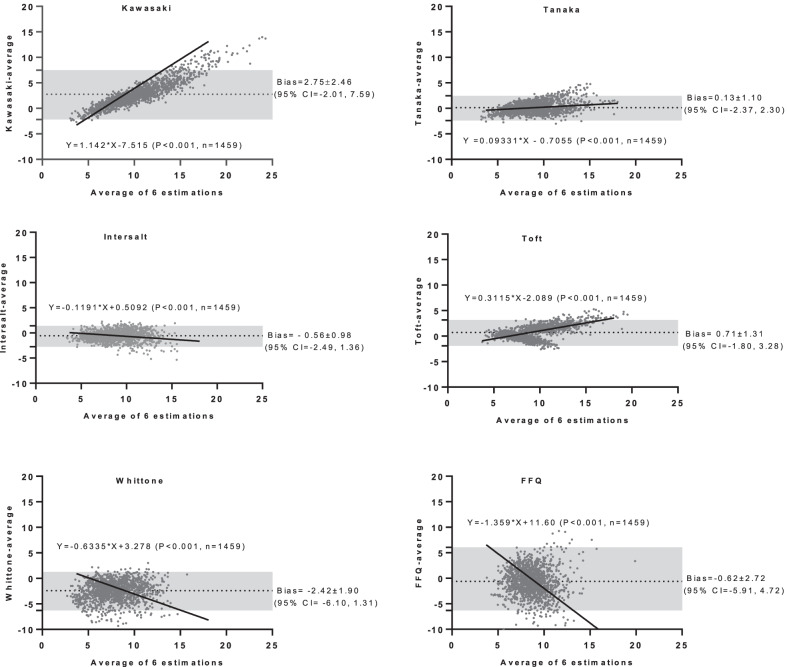
Mean bias, SD and 95% CI of each method compared to the gold-estimated salt intake (derived from the average of all methods), along with the slope and intercept for bias of each estimation *vs.* overall mean of 6 estimations

Mean (SD) estimated salt intake, derived from the overall mean of the methods, was 9.0 ± 2.2 g/d (10.2 ± 2.1 and 7.9 ± 1.7 in men and women, respectively). Compared with the gold-estimated salt intake, derived from the average of all methods, the mean bias ranged from − 2.42 to 2.75 g/d, with the Tanaka equation, had the least bias (mean bias = 0.13 ± 1.10, 95% CI − 2.37, 2.30 g/d) and the Kawasaki (mean bias = 2.75 ± 2.46, 95% CI − 2.01, 7.59 g/d) and Whitton (mean bias = − 2.42 ± 1.90, 95% CI − 6.10, 1.31 g/d) equations had the most bias (Fig. 1).

The slope and intercept for bias of each estimation *vs.* the overall mean of 6 estimates, obtained by the least squared perpendicular distance regression analysis, are reported in Table 3. The table also shows the least-biased point of each equation, i.e., the mean bias of the estimation to the overall mean was tended to be zero. The values showed that the most accurate equations for low- and high-salt intake levels are Intersalt and FFQ, respectively (least-biased point = 4.3 and 8.5 g/d salt intake). The least-biased point for the Tanaka equation was 7.6 g/d. The difference to overall mean at the low, medium, and high-salt intake for the estimations are provided in Table 3. The Tanka equation showed a mean bias < 1 g/d (range − 0.2 to 0.7 g/d) at all levels of salt intake. The Intersalt underestimated salt intake at low-, medium-, and high-level of salt intake, while Kawasaki, Toft, and Tanaka underestimated at low intake (< 5 g/d) and overestimated at high intake (> 15 g/d). Inversely, the Whitton equation and FFQ overestimated at low intake and underestimated at a high level of salt intake.

The Tanaka, the most fitted equation for salt estimation among our population, estimated a mean salt intake of 8.9 g/d (9.5 and 8.3 g/d, in men and women, respectively) with a range of 2.1 to 18.7 g/d. Only 5.1% (1.9% and 7.7% in men and women, respectively) adhered to WHO recommendation (< 5 g/d salt intake), whereas 26.8% (33.5% and 21.5% in men and women, respectively) and 2.4% (3.0% and 1.9% in men and women, respectively) exceeded the recommendation by more than 2- and threefold. About 6.6%, 12.0%, 15.4%, 16.9%, and 14.8% of the population exceeded the recommendation of salt intake by 1, 2, 3, 4, and 5 g/d, respectively.

## Discussion

Our study indicated that the Tanaka equation, conducted based on urinary Na, K, and Cr concentrations from casual urine specimens may be a helpful method for estimating mean 24-h Na excretion and population salt intake. Compared with the gold-estimated salt intake, i.e., derived from the average of urine-based and FFQ-based procedures, the Tanaka equation provided the least biased estimation with a mean bias < 1 g/d at low-, medium-, or high-level of salt intake among our population. Population salt estimation using the Tanaka equation showed that most of the Iranian population (about 95%) exceeded the current recommended limits of salt intake.

The performance of usual equations used for 24-h Na excretion from spot urine samples has remained inconsistent among different populations [19]. The estimated 24-h urinary Na using the usual equations (i.e., Kawasaki, Tanaka, and Intersalt) were reported to be systematically biased with overestimation at lower levels and underestimation at higher levels of Na intake; other variables in the equations seem to contribute to the biased estimates since a similar systematic bias occurred where Na concentration was kept constant (41). Similar to our observation, the Kawasaki equation overestimates mean Na intake among several populations [41–43]. The Intersalt was more accurate than other equations estimating salt intake among different populations [41]. Dietary methods are also used to estimate population salt intake; however, they tend to underestimate Na intake due to the under-reporting of discretionary sources of salt [19, 21].

In our study, about 5.1% met salt intake recommendations, and about 26.8% and 2.4% exceeded by more than 2- and 3-fold. A recent national report of estimated Iranian salt intake indicated a mean population of 9.52 g/d (95% CI 9.48–9.56) and ~ 97.7% overconsumption of salt among the population [44].

It remains disputable whether there is a “healthy” or “normal range” of Na intake [45]; a range of 0.5–1 [46] up to 55 [47] g/d salt intake was reported (based on urinary Na excretion) among populations. The WHO technical report on “Reducing Salt Intake in Populations” in 2006 represents a wide range of 24-h urinary Na (from 0.8 to 299 mmol/d) among different populations; this report indicates that most people appear to have mean 24-h urinary Na over 100 mmol/d, and among the Asian countries this value exceeded 200 mmol/d [48]. Combining 197 datasets (69 799 subjects) over a 3-decade period indicates that human Na intake follows a classic normal distribution that depicts a narrow range with strict lower and upper limits of normality (mean = 159.2, range 90–248 mmol/d; mean after removal of outliers = 159.4, range 114–210 mmol/d) [17].

The WHO Global Non-communicable Diseases Action Plan 2013–2020 sets a 30% relative reduction in the mean population Na intake by 2025 [49]. Despite 4-decade comprehensive public policies, the distribution of population Na intake has not changed over time [17], and long-term low-Na-diet trials (85 mmol/d Na) could not achieve a significant reduction in Na intake [50]. Meta-analysis of both randomized clinical trials and observational studies resulted in a weak association between salt intake and BP, especially among non-obese individuals with normal BP [51, 52]; an effect size of 1 mm Hg in BP following a low-Na diet does not justify a general restriction for Na intake [52]. On the other hand, a salt intake lower than 5.8 g/d was reported to be associated with the activation of the renin–angiotensin–aldosterone system, increased plasma lipids, and increased mortality [51]; risk of myocardial infarction, cardiovascular diseases, and all-cause mortality was increased among hypertensive patients with increased plasma renin activity and low-urinary levels of Na [53, 54]. Furthermore, a meta-analysis of population Na intake showed a "U shape" relationship with the risk of mortality [55], and mean estimated Na intake was inversely associated with mortality at a level of < 4 g/d (10 g/d salt) [41]. Previous studies among our population also showed no significant association between dietary intake of Na and the risk of hypertension, CVD, and renal dysfunction [56, 57]. Current evidence relating Na intake to hypertension and CVD has significant limitations [9] and could not provide a strong statement on the adverse effect of Na intake on CVD outcomes and all-cause mortality [58].

Na intake appears to be set by human physiology, maintaining a minimal Na intake close to the lower limit of the normal range and approaching the upper limit obtained by global estimation of urinary Na excretion [45]. Such evidence may call for revisiting the dietary salt guidelines and maintaining optimal Na intake within the normal range identified by worldwide 24-h urinary Na surveys of populations [51].

Although it can be different among populations, about 75% of dietary Na is attributed to processed foods, 10–12% is naturally occurring in foods, and the rest of 10–15% is discretionary salt intake (salt used in home-cooking or at the table) [48, 59]. Bread products, cereal, and grains have been responsible for about 40% of total Na intake [48]; meat and dairy products are the major contributors to dietary Na intake in most populations [60]. More than half of their daily salt intake is from discretionary sources in some countries (i.e., Brazil, China, Costa Rica, Guatemala, India, Japan, Mozambique, Romania) [60].

As a strength, this was the first study among an Iranian urban population with a relatively large sample size that estimated salt intake using several urine-based methods and an FFQ-based approach. Our study also had some limitations; first, due to the lack of 24-h urinary samples as the gold standard method for measuring Na's urinary excretion and salt intake, we used the overall mean of the estimations to assess the performance of equations. The second and the most critical limitation of the present study was that the equations used for salt estimation were initially developed and validated for other populations; we, therefore, need to establish a best-fitted equation for our population. Finally, FFQ provided the mean Na intake of the participants over the last year, however, spot urine sample possibly provided Na status of a single day, and therefore, FFQ seems to be different from a urine sample in estimating Na intake.

## Conclusion

In conclusion, our study also showed that about 95% of the Iranian population exceeded current salt intake recommendations. The study also indicated that the Tanaka equation might be the best model for population salt intake using spot specimens among our people. Since the performance of casual urine samples for estimating Na excretion was reported to be independent of the time of urine sample collection (i.e., overnight, morning, afternoon, and evening), this method seems to have priority as the best alternative of 24-h urine sample collection for estimating population salt intake.

**Table 1:** Commonly used equations for estimating 24-h urinary Na excretion from the spot urine sample

Name of equation	Description	History
Kawasaki (1993) [29]	Male = 23 × 16.3 × [0.1 × (15.12 × Weight + 7.39 × Height − 12.63 × Age − 79.9) × Spot Na ÷ Spot Cr]^0.5^ Female = 23 × 16.3 × [0.1 × (8.58 × Weight + 5.09 × Height—4.72 × Age − 74.5) × Spot Na ÷ Spot Cr]^0.5^	Initially developed among a Japanese population (159 healthy, free-living 20–79 y adults, using second morning voiding urine specimens and 24-h urine sampling (for 3–5 days)
Tanaka (2002) [30]	Both genders = 23 × 21.98 × (0.1 × [- 2.04 × Age + 14.89 × Weight + 16.14 × Height − 2244.45] × Spot Na ÷ Spot Cr)^0.392^	Initially developed among a Japanese population (591 adults, 20–59 y) using casual urine specimens and 24-h urine sampling
Intersalt (2013) [31]	Male = 23 × (25.46 + 0.46 × Spot Na—2.75 × Spot Cr − 0.13 × Spot K + 4.10 × BMI + 0.26 × Age) Female = 23 × (5.07 + 0.34 × Spot Na—2.16 × Spot Cr − 0.09 × Spot K + 2.39 × BMI + 2.35 × Age − 0.03 × Age^2^)	Developed among the North American and European populations (5693 adults aged 20–59 y)
Toft (2014) [32]	Male = 33.56 × [Spot Na ÷ Spot Cr × (− 7.54 × age + 14.15 × weight + 3.48 × height + 423.15]^0.345^ Female = 52.65 × [Spot Na ÷ Spot Cr × (− 6.13 age + 9.97 × weight + 1.24 × height + 342.73] ^0.196^	Developed among Danish individuals (473) using 24-h urine collection and a spot urine sampling
Whitton (2016) [20]	Both genders (for morning sample) = 23 × (88.66 + 0.55 × Spot Na—1.34 × Spot Cr—1.05 × Spot K − 0.87 × Age + 2.10 × BMI + 39.30 × Sex (male = 1, female = 0) + Ethnicity^†^ (Malay = − 17.70, Indian = − 10.38, Chinese = 0)	Developed among Singaporean or permanent residents of Singapore (144 subjects, aged 18–79 y) using multiple spot urine sampling (morning, afternoon, evening) and a 24-h urine sampling

Body weight in kg, BMI in kg/m^2^, spot Na in mmol/L, spot Cr in mmol/L, age in years, height in cm

^†^Considered similar to Chinese as 0

**Table 2:** Characteristics of the study participants

	Total (n = 2069)	Men (n = 940)	Women (n = 1129)
Age, y	45.6 ± 14.8	46.7 ± 15.7	44.6 ± 13.8^*^
BMI, kg/m^2^	27.9 ± 5.2	27.4 ± 4.4	28.2 ± 5.7^*^
Waist circumference, cm	93.5 ± 12.5	95.9 ± 11.6	91.5 ± 12.9^*^
Systolic blood pressure, mmHg	114 ± 17	119 ± 16	109 ± 17^*^
Diastolic blood pressure, mmHg	75.6 ± 10.5	78.2 ± 10.4	73.4 ± 10.2^*^
Casual urinary Cr, mmol/L	13.8 ± 6.1	14.2 ± 6.0	13.5 ± 6.2^*^
Estimated^†^ 24-h urinary Cr, mmol/d	13.0 ± 4.0	16.5 ± 3.3	10.0 ± 10.3^*^
Casual urinary Na, mmol/L	135 ± 56.5	143 ± 54.9	127 ± 56.8^*^
Casual urinary K, mmol/L	71.6 ± 36.2	72.8 ± 35.3	70.7 ± 36.9
Casual urinary Na-to-K ratio	2.28 ± 1.39	2.39 ± 1.45	2.19 ± 1.34^*^
FFQ-estimated^‡^ Na intake, mmol/d	144 ± 36.3	142 ± 37.3	145 ± 35.4
FFQ-estimated^‡^ K intake, mmol/d	109 ± 28.5	104 ± 28.6	112 ± 28.2^*^
FFQ-estimated Na-to-K ratio	1.48 ± 2.16	1.59 ± 3.14	1.38 ± 0.57
Energy intake, kcal/d	2300 ± 811	2501 ± 896	2134 ± 690^*^
Dietary protein, g/d	90 ± 41.6	98.4 ± 44.9	83.0 ± 37.2^*^
Dietary fat*, g/d*	75.7 ± 32.5	79.7 ± 36.7	72.4 ± 28.2^*^
Dietary carbohydrate, g/d	342 ± 125	376 ± 135	313 ± 108^*^
Dietary fiber, g/d	45.1 ± 22.3	49.3 ± 23.9	41.6 ± 20.2^*^
Physical activity, METs h/week	69.4 ± 42.6	85.6 ± 46.5	56.1 ± 33.8^*^
Current smoking, %	16.7	30.2	5.40^*^
Educational level			
Illiterate, %	1.50	1.00	2.00^*^
Diploma, %	59.5	58.6	60.3
Academic, %	40.5	41.4	39.7

^*^P < 0.05 compared to men using independent sample t-test or Chi-square was used

^†^Based on Kawasaki equation

^‡^Residual-energy adjusted

BMI, body mass index; FFQ, food frequency questionnaire; Na, sodium; K, potassium; Cr, creatinine

**Table 3:** The slope and intercept for bias of each estimation *vs.* overall mean of 6 estimations

Methods of estimation	Linear regression model		Zero difference to overall mean	Difference from overall mean at the low, medium and high salt intake (g/d)		
	Slope	Intercept		5	10	15
Kawasaki	1.14 (1.10, 1.18)	− 7.52 (− 7.91, − 7.12)	6.6	− 1.8	3.9	9.6
Tanaka	0.09 (0.06, 0.18)	− 0.71 (− 1.02,− 0.39)	7.6	− 0.2	0.2	0.7
Intersalt	− 0.12 (− 0.15, 0.09)	+ 0.51 (0.25, 0.76)	4.3	− 0.1	− 0.7	− 1.3
Toft	0.31 (0.27, 0.35)	− 2.09 (− 2.45, − 1.73)	6.7	− 0.55	1.0	2.6
Whitton	− 0.63 (− 0.73, − 0.54)	+ 3.28 (2.39, 4.16)	5.2	0.1	− 3.1	− 6.2
FFQ	− 1.36 (− 1.44, − 1.28)	+ 11.60 (10.88, 12.32)	8.5	4.8	− 2.0	− 8.8

## Data Availability

Data will be presented upon forwarding the request to the corresponding author (ghasemi@endocrine.ac.ir) and confirmation of the director of RIES (azizi@endocrine.ac.ir).

## Abbreviations

BMI: Body mass index
Cr: Creatinine
DALYs: Disability-adjusted life-years
DBP: Diastolic blood pressure
DGA: Dietary Guidelines for Americans
FFQ: Food frequency questionnaire
K: Potassium
Na: Sodium
OLR: Ordinary linear regression
SBP: Systolic blood pressure
SD: Standard deviation
TLGS: Tehran Lipid and Glucose Study
WHO: World Health Organization
